# Superomedial Pedicle Technique and Management of Circulation Problems in Gigantomastia: Treatment of Gigantomastia

**DOI:** 10.1007/s00266-024-03913-6

**Published:** 2024-03-20

**Authors:** Recep Anlatici, Sarp Demiralay, Omer Parildar, Omer Refik Ozerdem

**Affiliations:** 1 G.Antep University School of Medicine Head of Plastic and Reconstructive Surgery, G. Antep, Turkey; 2 Sade and Demiralay Clinic, Abdi Ipekci Cd. No:61, 34367 Sisli/İstanbul, Turkey; 3 G.Antep City Hospital, Consultant Plastic Surgeon, 27470 Şahinbey G. Antep, Turkey; 4 Professor Ozerdem Clinic, M.Kasapoglu Cd 1446 Sk B-blok Suite 14, Muratpasa, Antalya, Turkey

**Keywords:** Reduction mammaplasty, gigantomastia, Mammaplasty complications, Vacuum-assisted therapy, Hyperbaric oxygen therapy, Superomedial pedicle reduction mammaplasty

## Abstract

Breast reduction surgeries encompass a wide range of methods that are continuously evolving to discover more reliable and satisfactory techniques. This presentation aims to address the research gap by sharing outcomes and experiences using the superomedial pedicle in gigantomastia, as well as the implemented protocol for managing nipple-areola complex (NAC) ischemia. The Wise pattern and superomedial pedicle reduction mammaplasty method were utilized in treating 19 patients (38 breasts). The average age of the patients was 41.47 years, with a basal mass index (BMI) of 33.27 kg/m^2^. The mean sternal notch to nipple (SN-N) length for the entire population was found to be 40.97 cm. On both sides, this length was statistically similar at 41.11 cm on the right side and 40.84 cm on the left side. The average weight of resected tissue from all patients was calculated to be 1793.42 g, with slightly higher weight on the right side at 1800 g compared to the left side’s weight of 1786.84 g. Postoperative NAC ischemia occurred in three patients, one bilateral case, and two unilateral cases. The study revealed that in both the groups with and without NAC ischemia, the average values were as follows: age, which ranged from 45.33 to 40.75 years; BMI, ranging from 35.01 kg/m^2^ to 32.95 kg/m^2^; SN-N distance, which varied from 40 cm to 41.09 cm; and excision material weights, ranging from 1650 g to 1810.29 g. The p-value in the comparisons was found to be greater than 0.05. These results indicate that age, BMI, SN-N distance, and excision material weight did not have an impact on NAC vascularity issues. All NACs were successfully saved through a protocol involving hyperbaric oxygen therapy (HOT) and vacuum-assisted therapy (VAT). The study suggests that utilizing a superomedial flap is a viable option for treating gigantomastia and highlights the effectiveness of their outlined protocol in managing postoperative complications. While acknowledging the need for comparative studies, the study proposes incorporating HOT and VAT into protocols aimed at saving NACs.

*Level of Evidence IV* This journal requires that authors assign a level of evidence to each article. For a full description of these Evidence-Based Medicine ratings, please refer to the Table of Contents or the online Instructions to Authors www.springer.com/00266.

## Introduction

Breast reduction operations constitute a diverse field that continually evolves to discover more reliable and satisfying methods [1–17]. These operations are known to enhance an individual’s daily life by alleviating symptoms such as back and shoulder pain and potentially reducing the risk of breast cancer [1]. While there is no universally agreed upon weight criterion for excision material per breast to define gigantomastia in the literature (some authors set the lower limit at 1000 g., while others opt for 1500 g.), it is evident that gigantomastia cases present unique challenges concerning health issues related to breast size, the selection of treatment methods, and concerns about postoperative complications [6, 9, 18]. Despite the ongoing quest for safer gigantomastia treatment methods, the scarcity of publications addressing the management of potential complications during its treatment remains a noteworthy challenge [19, 20]. With this presentation, our objective was to address the relative lack of research in the field, sharing the results and experiences from our practices utilizing the superomedial pedicle in gigantomastia. Additionally, we aimed to present the protocol we follow when encountering nipple-areola complex (NAC) ischemia.

## Materials–Methods

The wise excision pattern was employed in our cases. A superomedial full-thickness flap with a base-to-length ratio of 1/3, carrying the NAC, was prepared, and rotated to its new position after completing the excision procedure. If necessary, only the distal part of the flap was elevated off the fascia to facilitate rotation. The angle of divergence of the skin flaps was set between 80 and 100 degrees, and the length of the vertical limbs at 6-8 cm. The new NAC placement was marked slightly below the level of the submammary sulcus, which generally corresponded to 23-24 cm as the sternal notch to nipple (SN-N) measurement in most patients.

The protocol we followed in cases where we detected or suspected postoperative NAC circulation problem was as follows:

- 1. No hesitation to open the sutures,
- 2. Cortisone injections to the pedicle (initiate with a total dose of 4 mg per breast qDay dexamethasone, then taper the dose to discontinue administration within a week or when deemed appropriate),
- 3. Topical nitroglycerin gel application on the NAC (5 mg per breast q12hrs, discontinue when deemed appropriate),
- 4. Hyperbaric oxygen therapy (HOT, a single 120-minute session per day at 2-3 ATA pressure for a period of 7 days, where 1 ATA= atmosphere absolute= 760 mmHg)
- 5. Control and monitoring of systemic health problems such as hypertension and diabetes
- 6. Low-molecular-weight heparin (enoxaparin 30-40 mg SC q12-24hrs, discontinue when deemed appropriate)
- 7. Add vacuum-assisted therapy (VAT) when needed.

## Statistical Analysis

The IBM SPSS Statistics for Windows, Version 25.0 (IBM Corp., Armonk, USA, 2017) package program was used to carry out the analyses. The compliance of numerical variables with normal distribution was assessed using the Shapiro–Wilk Test, employing the ANOVA model appropriate to the data structure, and utilizing error estimates obtained after analysis. Age and BMI were compared between two groups (with or without ischemia) using an Independent Two-Group Student t-Test. A randomized block design was employed to compare SN-N and tissue weight, with adjustment made for case and side (RXL) variables when comparing the two groups. Differences between categorical variables were examined using Fisher’s exact test. All hypothesis tests were conducted at a significance level of 0.05.

## Results (Figs. 1, 2, 3, 4, 5, 6, 7, 8, 9, 10, 11 and 12)

**Fig. 1:**
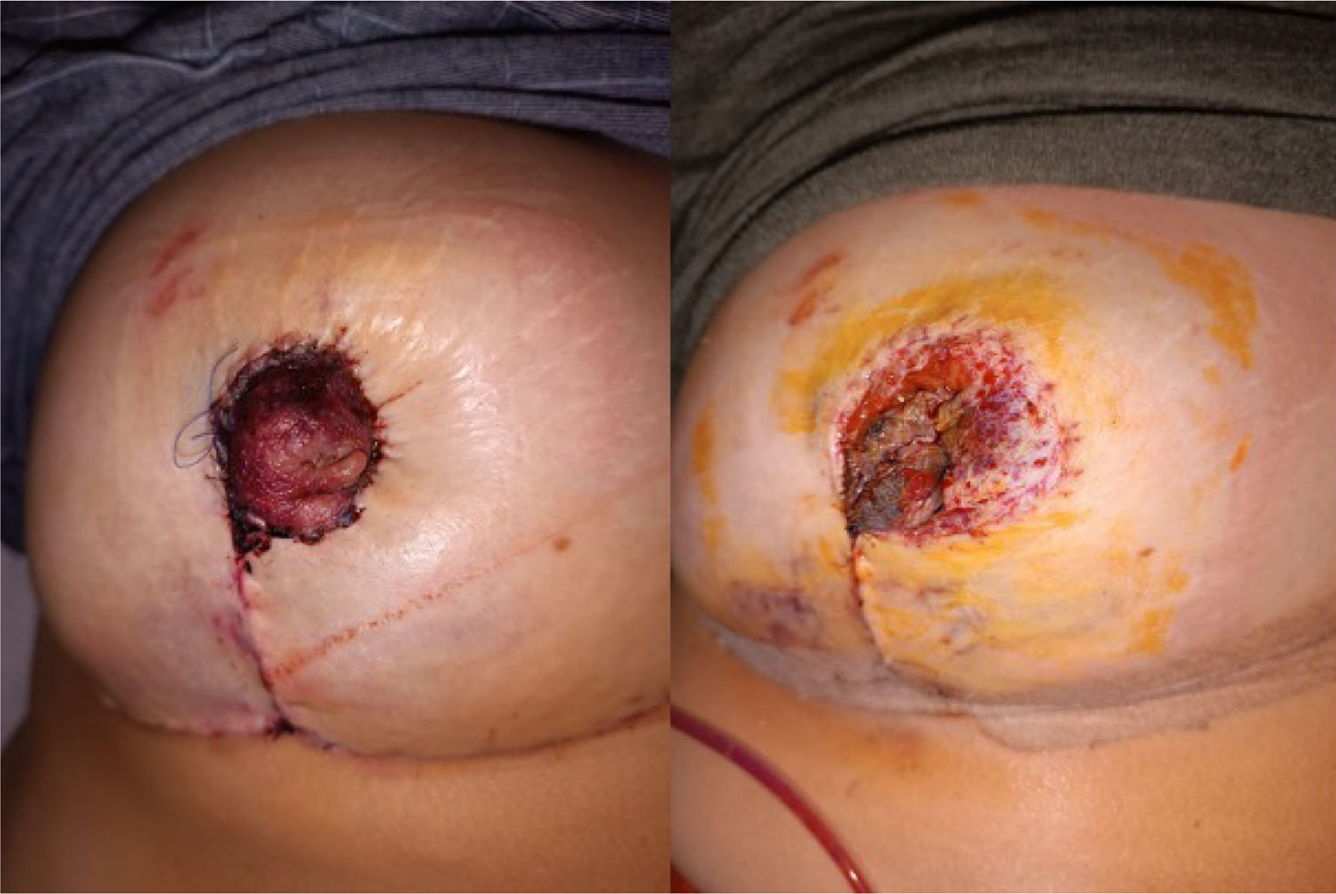
The right ischemic NAC detected in the 7th case in the early postoperative period, before and after stitch removal

**Fig. 2:**
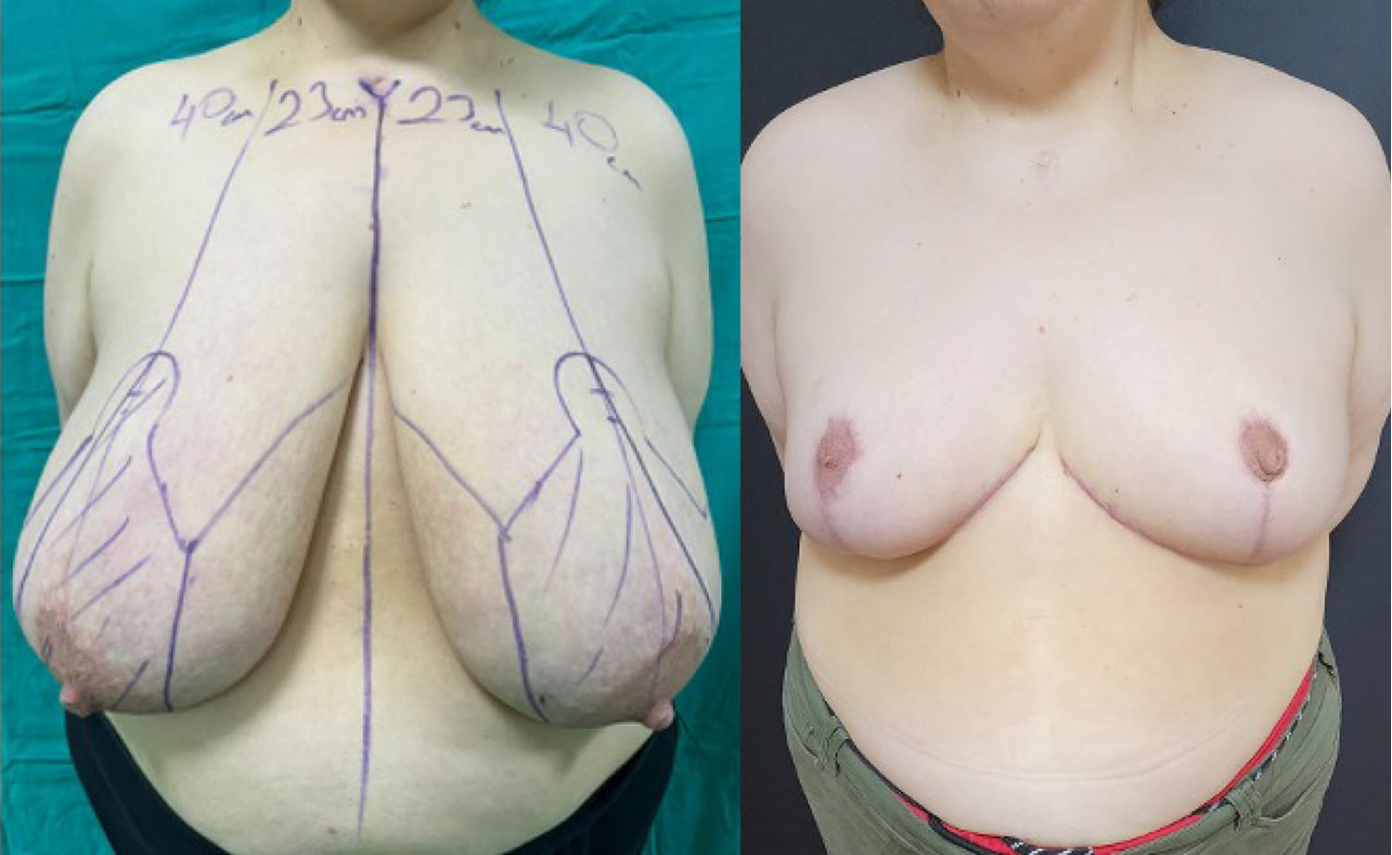
Front-view, preoperative and 6th month postoperative photographs of the case in Fig. 1

**Fig. 3:**
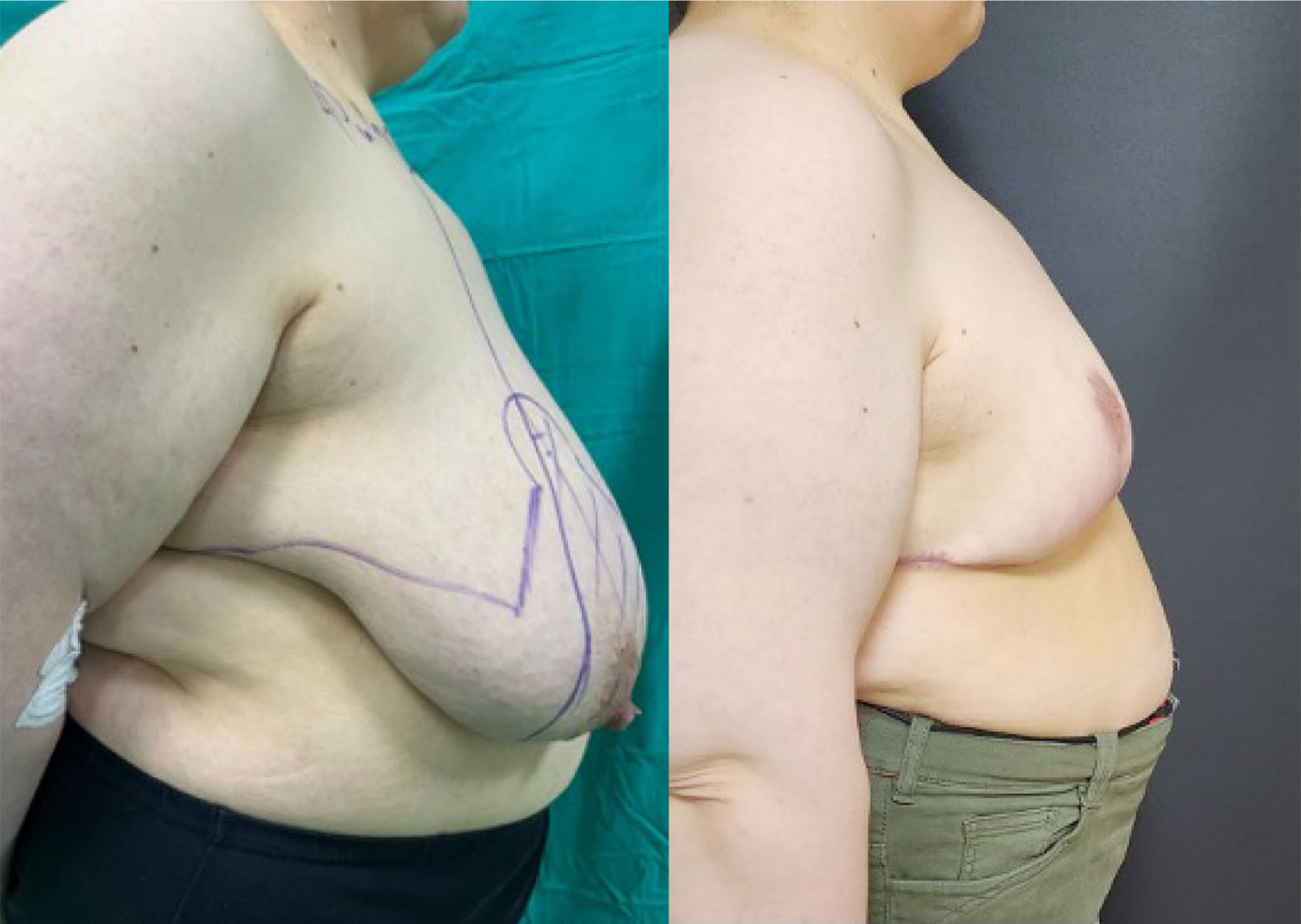
Right side view, preoperative and 6th month postoperative photographs of the case in Fig. 1

**Fig. 4:**
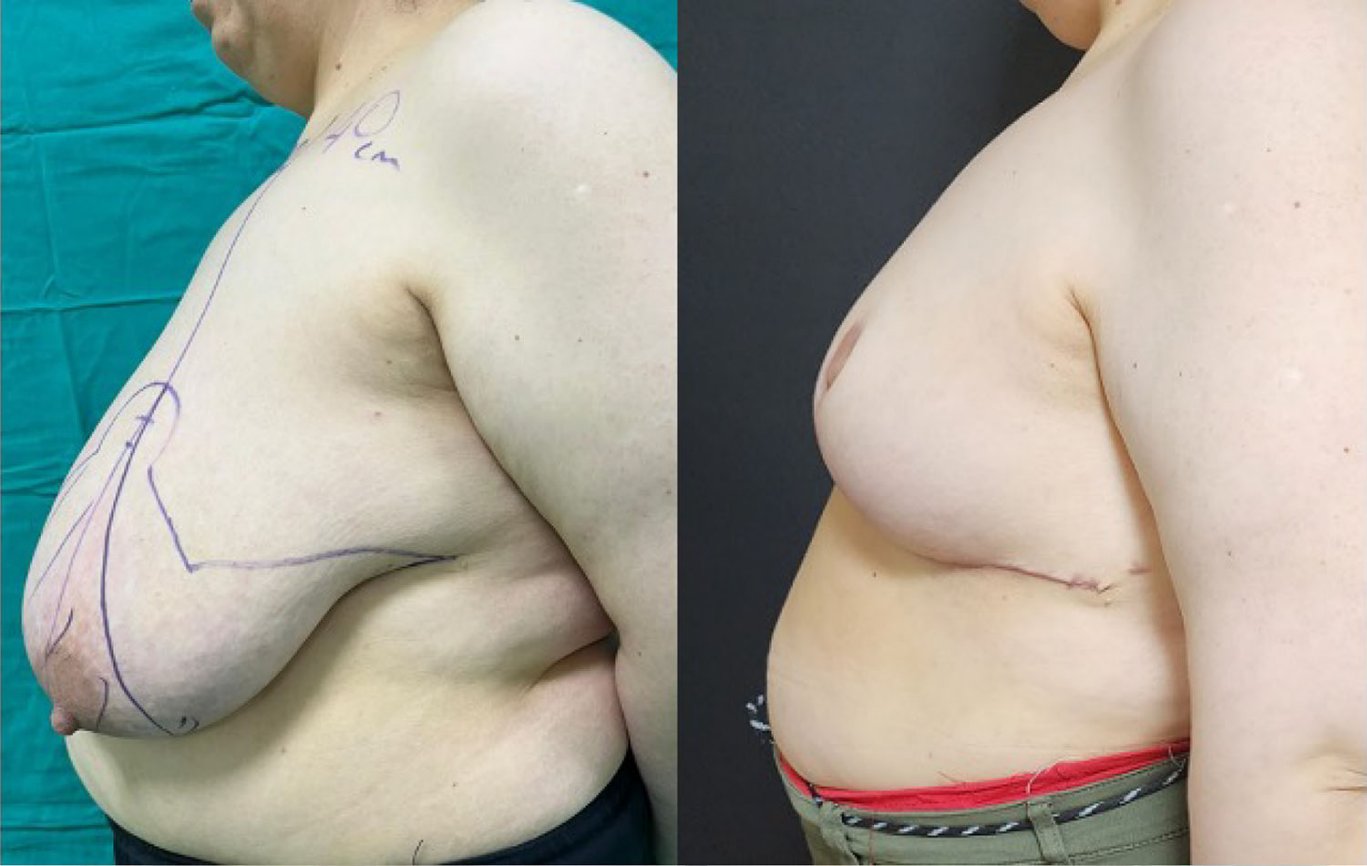
Left side view, preoperative and 6th month postoperative photographs of the case in Fig. 1

**Fig. 5:**
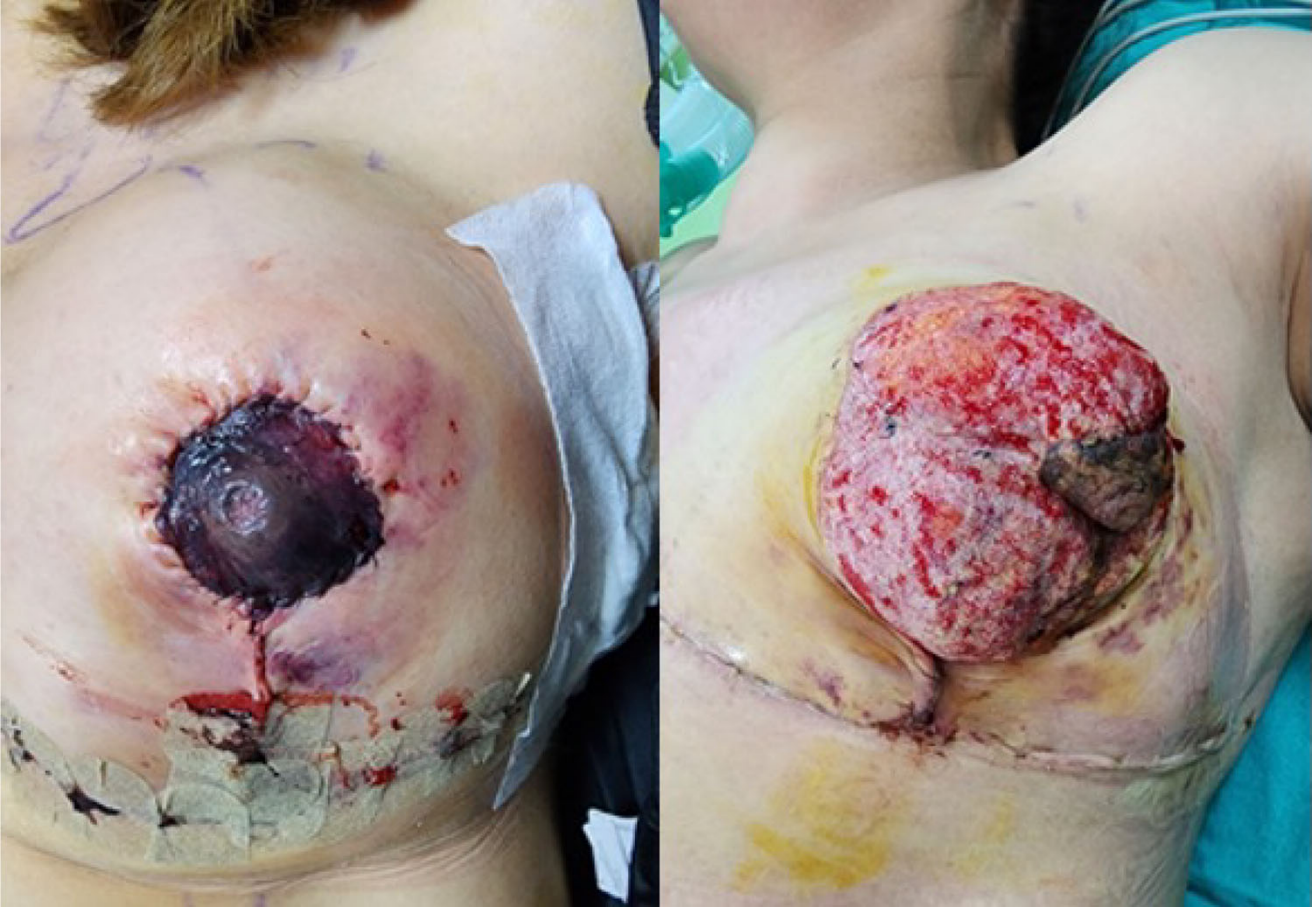
The left ischemic NAC detected in the 12th case in the early postoperative period, before and after stitch removal

**Fig. 6:**
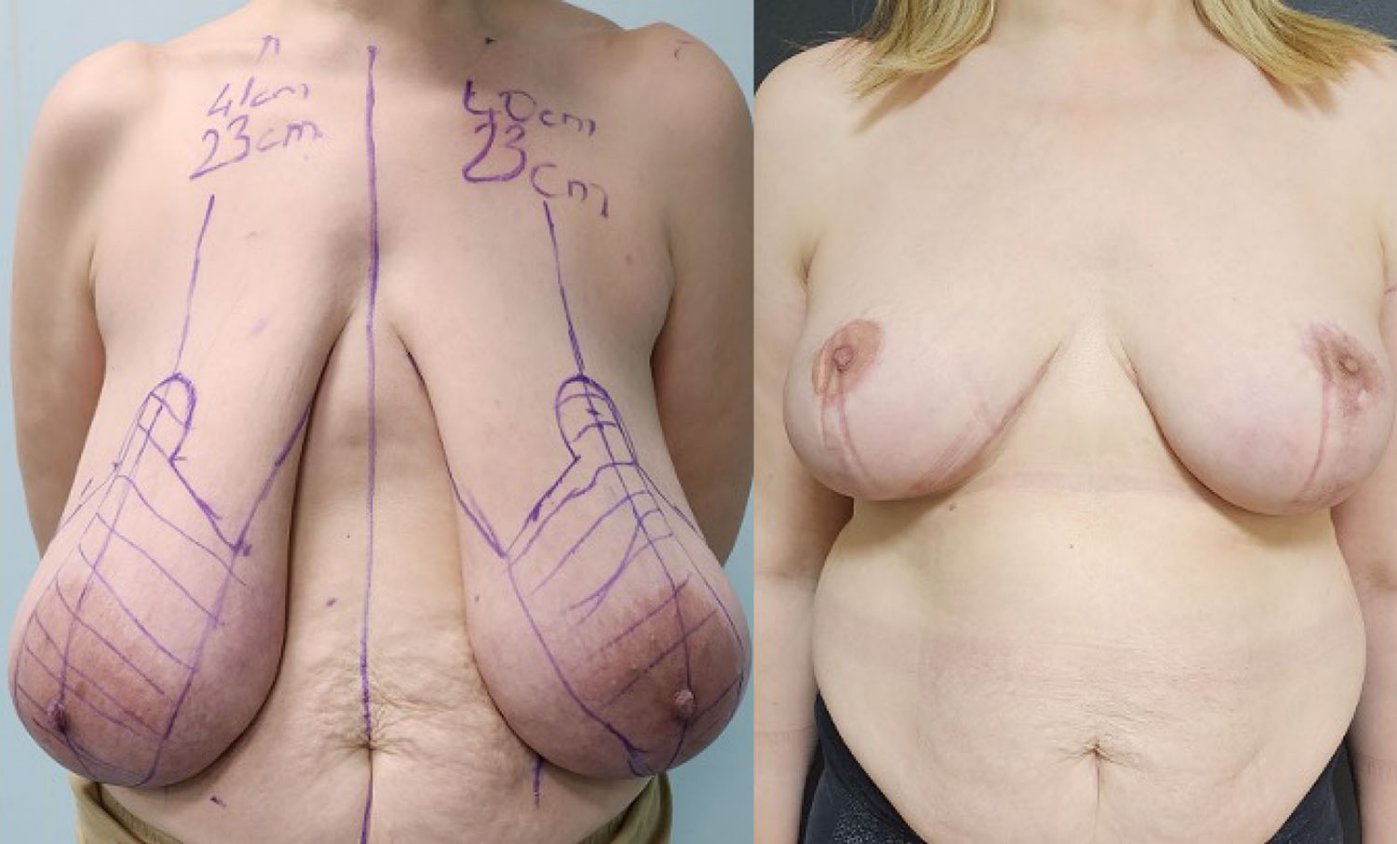
Front-view, preoperative and 7th month postoperative photographs of the case in Fig. 5

**Fig. 7:**
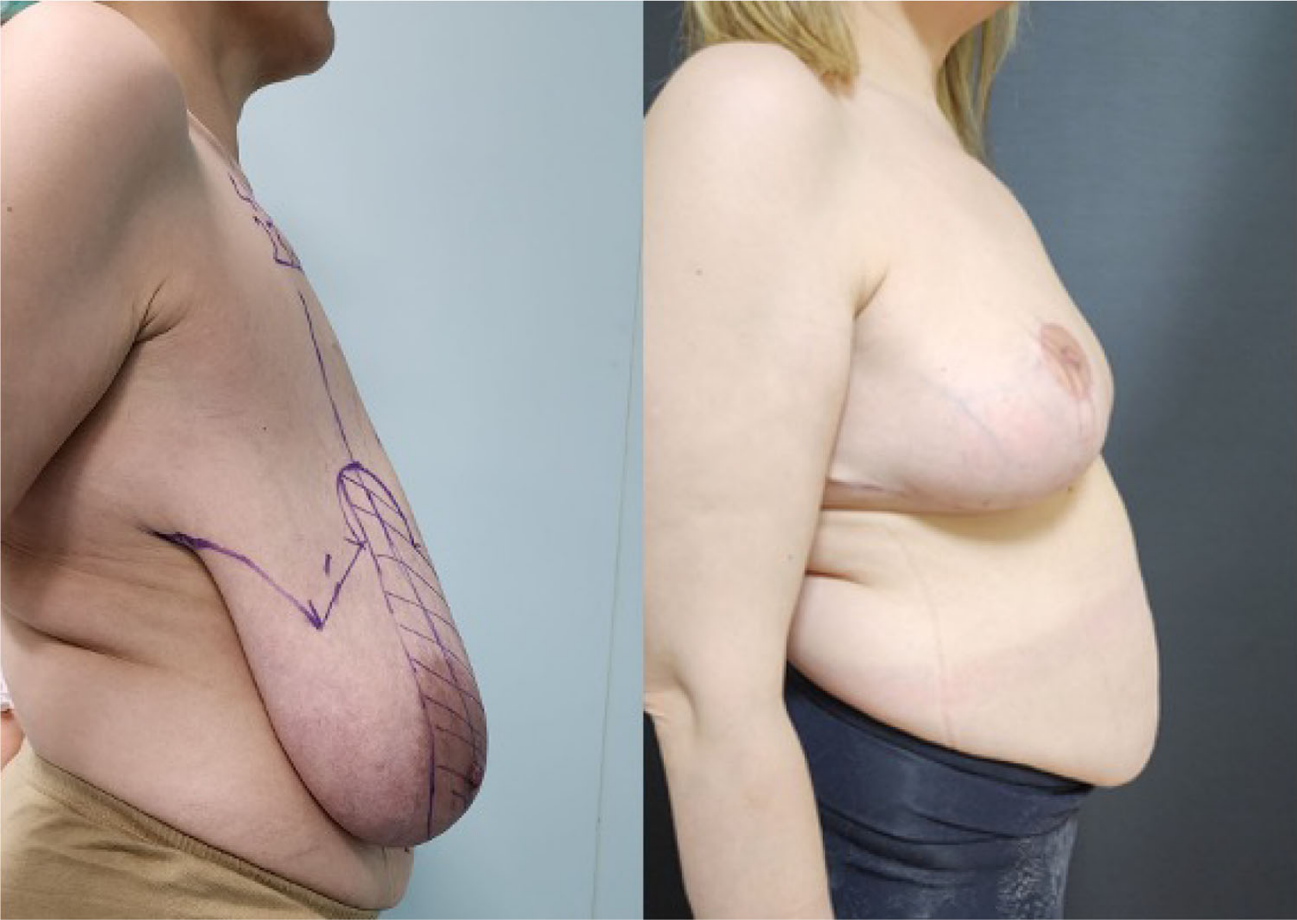
Right side view, preoperative and 7th month postoperative photographs of the case in Fig. 5

**Fig. 8:**
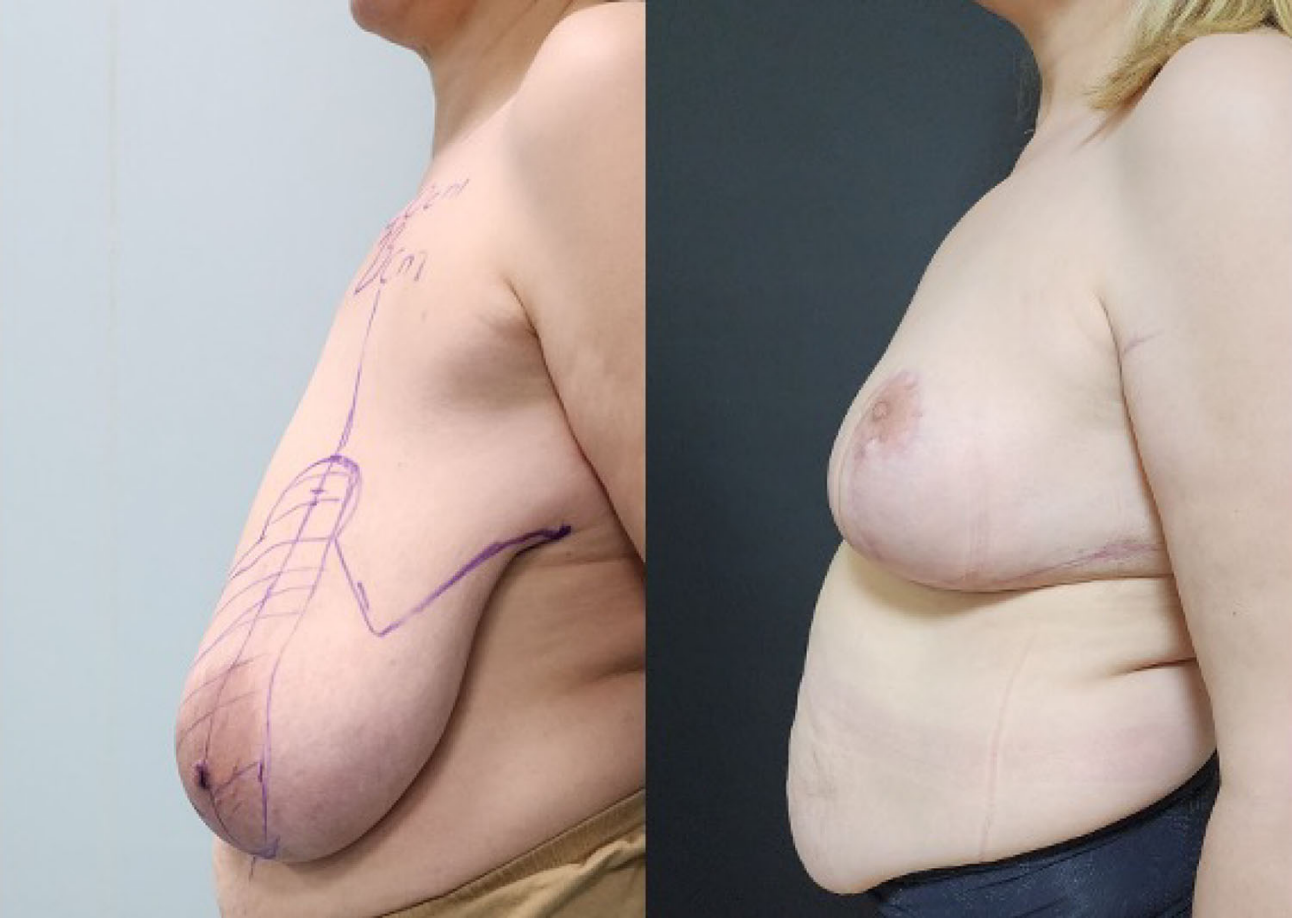
Left side view, preoperative and 7th month postoperative photographs of the case in Fig. 5

**Fig. 9:**
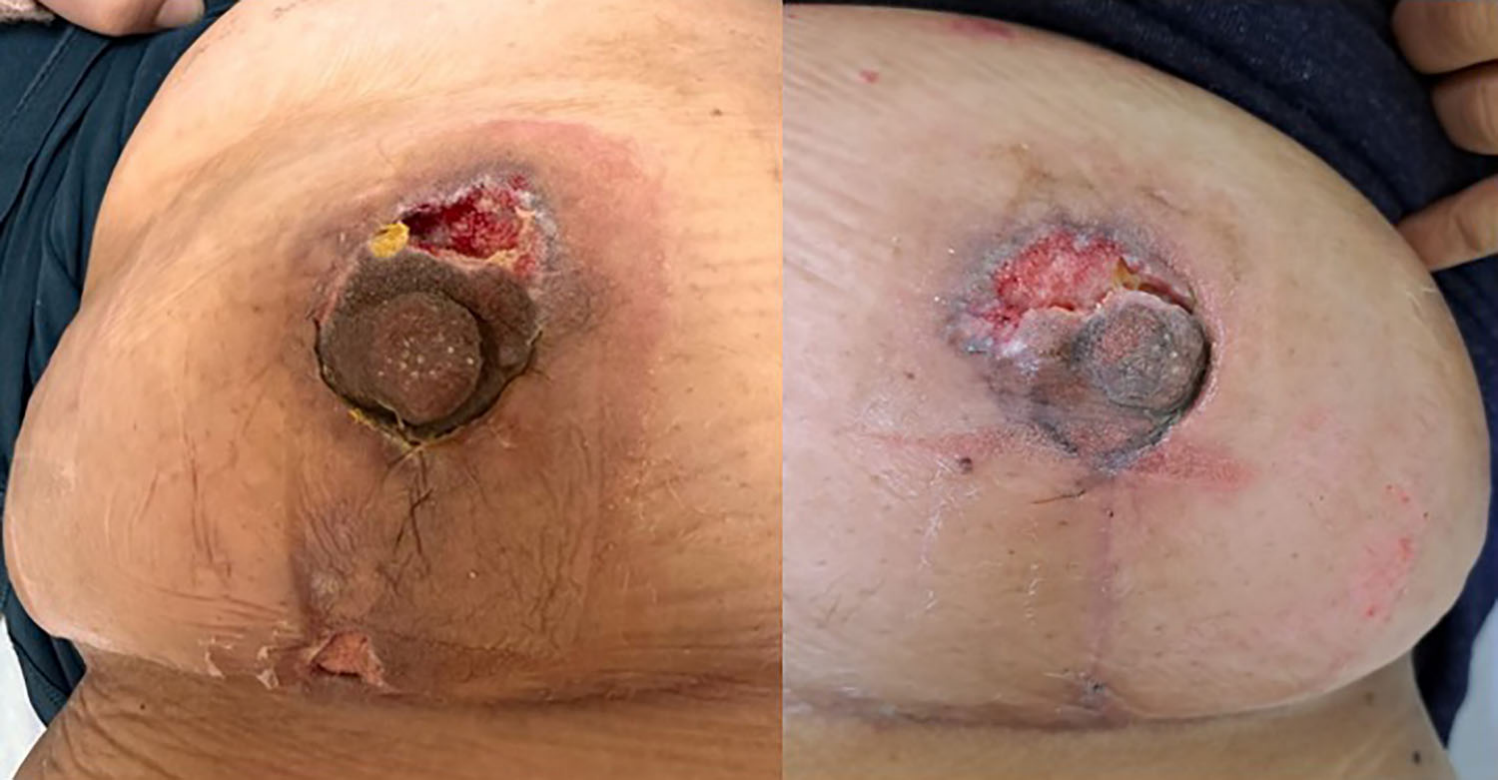
The appearance of the wounds during the 3rd postoperative week in case 1 involving bilateral partial NAC ischemia and fat necrosis. Both VAC and HOT treatments were applied to this case

**Fig. 10:**
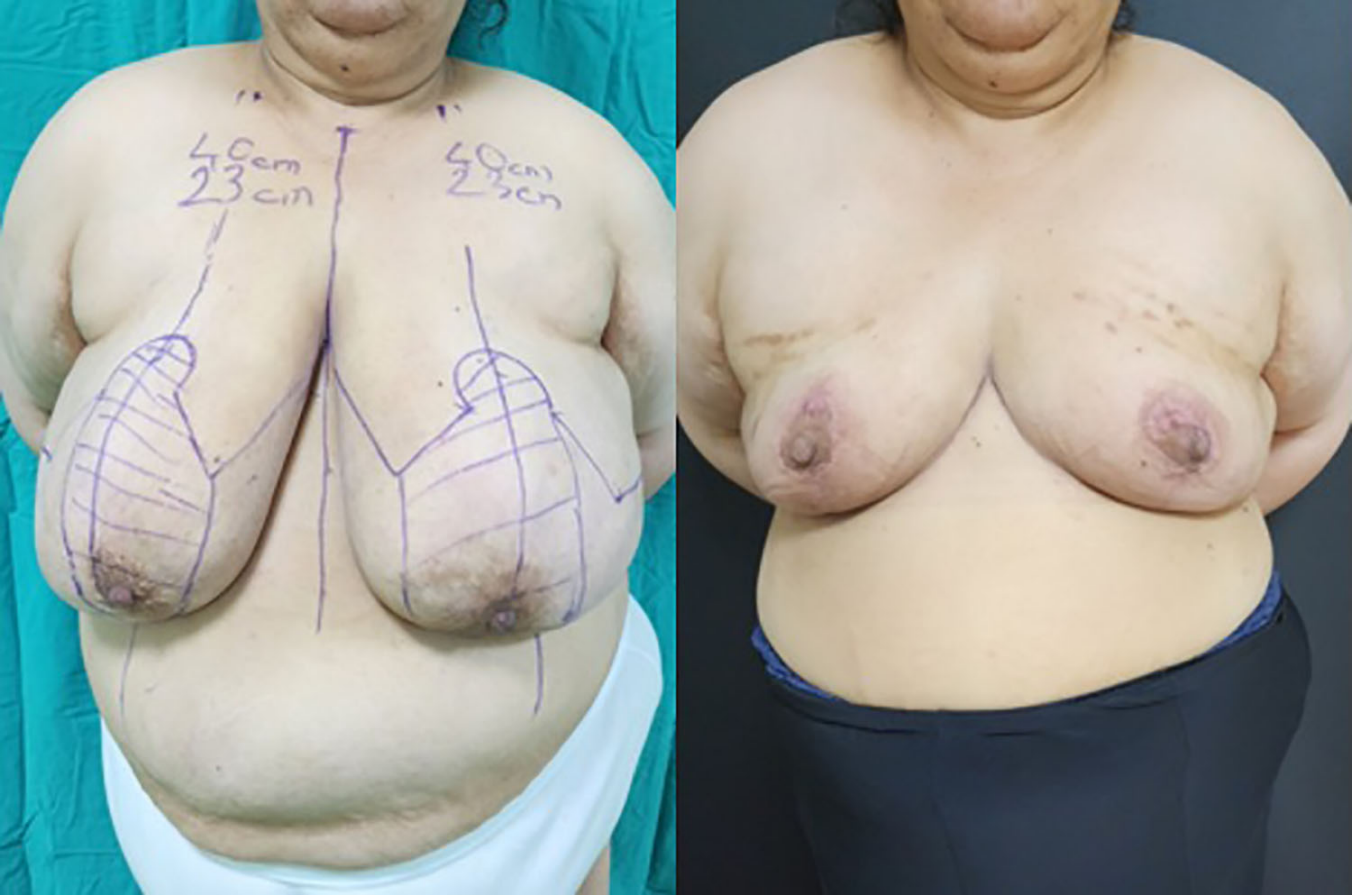
Front-view, preoperative and 5th month postoperative photographs of the case in Fig. 9

**Fig. 11:**
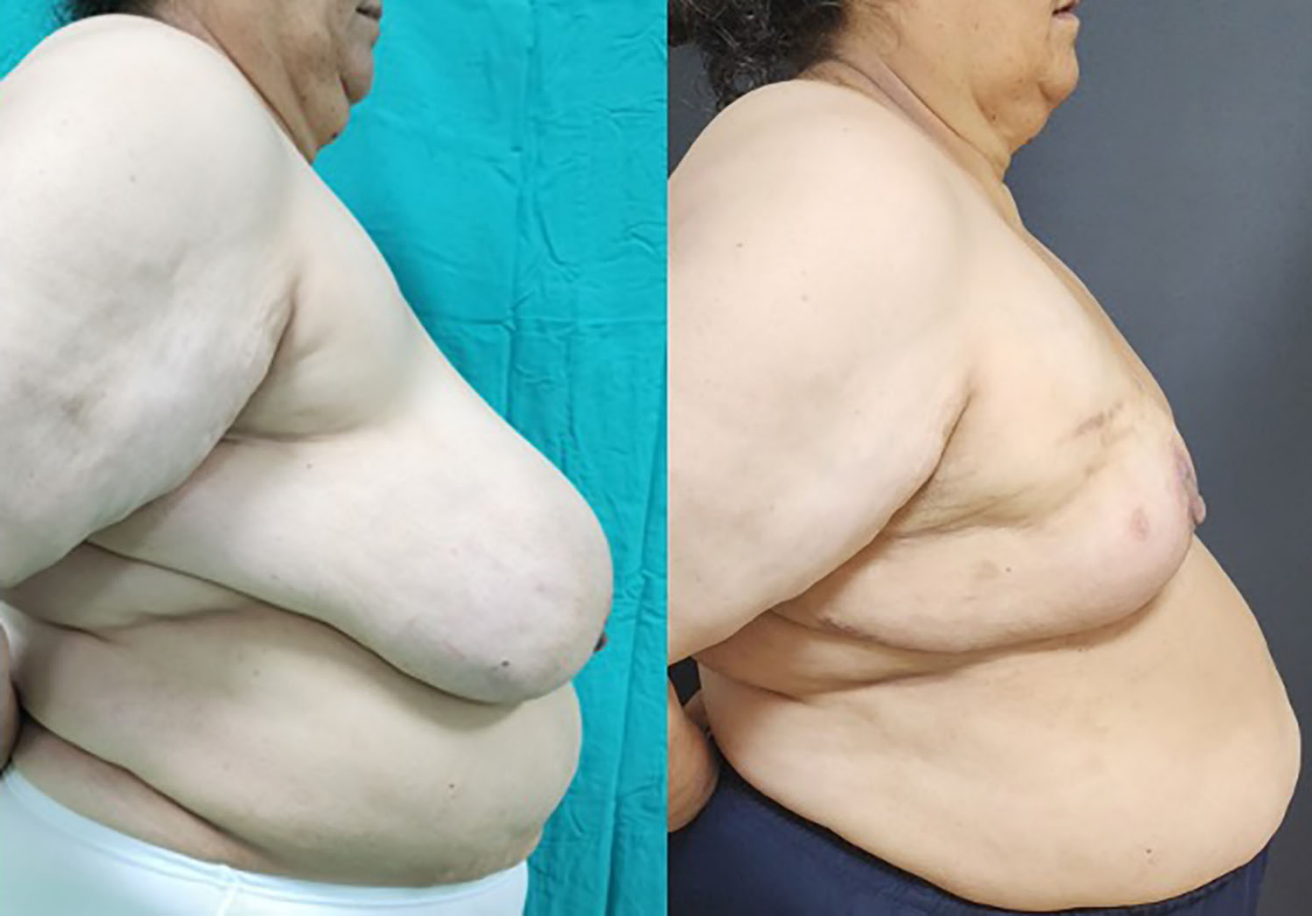
Right side view, preoperative and 5th month postoperative photographs of the case in Fig. 9

**Fig. 12:**
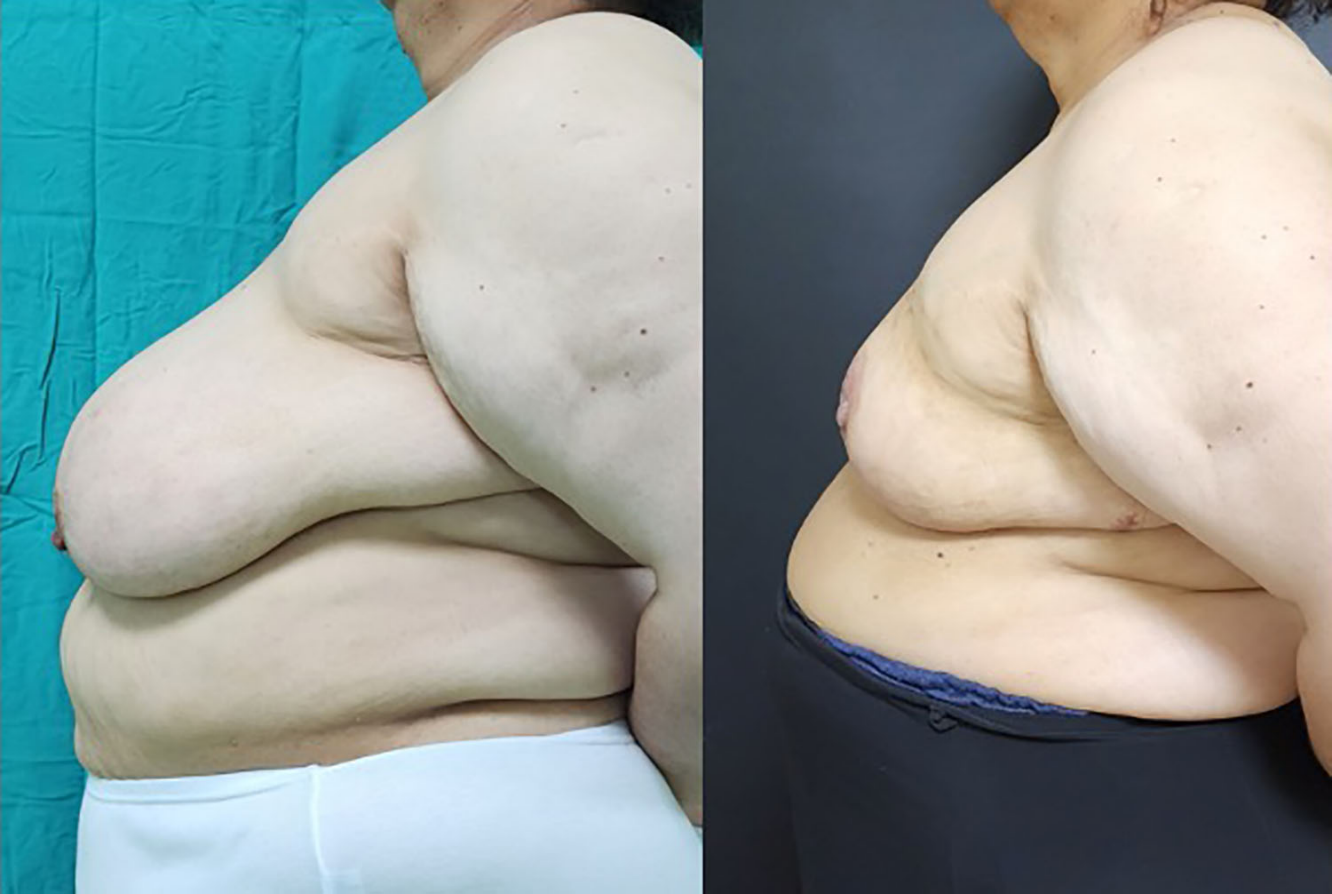
Left side view, preoperative and 5th month postoperative photographs of the case in Fig. 9

Our study included 19 patients with bilateral gigantomastia, resulting in a total of 38 breasts. We performed superomedial pedicle reduction mammaplasty and followed a specific protocol for cases of postoperative NAC ischemia over the past 5 years. The patients were monitored for a duration of 2 to 47 months, with an average of 13.32 months. Table 1 shows that the average age of the patients was 41.47 years, and their basal mass index (BMI) was 33.27 kg/m2. The mean SN-N length for the entire population was found to be 40.97 cm. On both the right and left sides, this length was statistically similar at 41.11 cm and 40.84 cm, respectively. The average weight of resected material from all patients was calculated to be 1793.42 g, with the right side being slightly higher at 1800 g compared to the left side’s weight of 1786.84 g.

**Table 1:** Characteristics of the study population

CASE#	AGE	BMI	SMOKING	R SN-N	L SN-N	R gr	L gr	Ischemia
1	51	39	No	40	40	1750	1750	Bil partial NAC ischemia & fat necrosis
2	25	32.17	1 pack	40	39	1500	1500	No
3	37	34.4	No	43	43	1850	1850	No
4	55	39.7	No	43	45	1500	1650	No
5	28	28.36	No	40	41	2100	2200	No
6	58	32	No	40	40	1900	1900	No
7	42	39.8	No	40	40	2000	2000	R complete NAC ischemia
8	53	33	No	42	40	2200	2000	No
9	51	36	No	40	42	2150	2250	No
10	47	29.8	No	40	39	1700	1650	No
11	37	27.5	No	40	40	1250	1250	No
12	43	26.23	1 pack	41	40	1150	1100	L complete NAC ischemia
13	39	35.46	1.5 pack	47	45	2300	2200	No
14	56	35	No	41	38	1650	1500	No
15	25	31.25	No	41	41	2000	2000	No
16	24	37.5	No	40	40	1500	1500	No
17	28	35	No	40	41	1800	1900	No
18	51	31	No	41	40	1900	1800	No
19	38	29	No	42	42	2000	1950	No
MEAN	41.47	33.27		41.11	40.84	1800	1786.84	

(SN-N: Substernal notch-nipple distance in cm; NAC: Nipple-areola complex; R: Right; L: Left; Bil: Bilateral. R gr or L gr: Weight of excision material in gr)

We did not observe any seroma or serious wound healing problems. Out of these cases, one involved partial ischemia in both NACs, while the remaining two were unilateral and completely affected (10.53% of all breasts affected). This resulted in a total of four affected breasts **(**Figs. 1, 5, 9**)**. This resulted in a total of four affected breasts. The patients experiencing NAC ischemia were identified as Case-1, Case-7, and Case-12, all with a common SN-N length ranging from 40 to 41 cm. Ischemic findings were detected in all cases within the first 12 hours. Subsequently, HOT treatment was initiated within the same timeframe. In the case with bilateral ischemia (case 1), additional findings such as exudation, local skin hyperemia and discoloration of the local fat tissue on the distal part of the superomedial flap were observed on the 3rd day, which prompted the immediate inclusion of VAC treatment. Debridement was not conducted for this case. The treatment protocol led to the preservation of NAC in all cases and the spontaneous healing of the wound and fat necrosis in case 1.

The angle between the vertical limbs of the skin flaps was initially set between 80 and 100 degrees, with lengths ranging from 6 to 8 cm, as stated before. However, all 3 patients with NAC ischemia and fat necrosis were among the initial 6 surgeries, with wise pattern limb lengths and divergent angles adjusted to around 6 cm and 100 degrees. Subsequently, the arms of the wise model were extended to 7-8 cm, and divergent angles narrowed to around 80 degrees for later applications, and no ischemia problem was diagnosed in the remaining patients. A significant statistical difference in the rates of NAC ischemia and fat necrosis was observed between the first 6 and the last 13 patients (50% to 0%, p < 0.05).

Out of the entire study group consisting of 19 patients, three individuals were smokers (accounting for 15.79%). However, among those who experienced NAC ischemia, the smoking rate increased to 33.33% (1 out of 3).

The comparative analysis between the group with NAC ischemia and the group without any problems yielded the following results (Table 2, p > 0.05 for all comparisons):

- The smoking rate in the study population was 3 out of 19, with a rate of 1 out of 3 in the NAC ischemia group and 2 out of 16 in the remaining group.
- The average age of the study population was 41.47 years. In the NAC ischemia group, it was 45.33 years, while in the non-problematic group, it was 40.75 years.
- The average BMI of the study population was 33.27kg/m2, with a higher figure of 35.01kg/m2 in the NAC ischemia group and a lower figure of 32.95kg/m2 in the non-problematic group.
- The average SN-N length for the study population was measured at 40.97 cm, with a slightly lower figure of 40 cm in the NAC ischemia group and a slightly higher figure of 41.09 cm in the non-problematic group.
- The average excision weight of the study population was 1793.47 grams, with a figure of 1650 grams in the NAC ischemia group and a higher figure of 1810 g in the non-problematic group.

**Table 2:** Statistical comparison between groups with and without NAC ischemia detected

Characteristics	NAC ischemia	No ischemia	General
Smoking, *n* (%)			
YES	1 (33.33%)	2 (12.5%)	3 (15.79%)
NO	2 (66.67%)	14 (87.5%)	16 (84.21%)
AGE, mean	45.33	40.75	41.47
BMI kg/m2, mean	35.01	32.95	33.27
SN-N cm, mean	40	41.09	40.97
Excision gr, mean	1650	1810.29	1793.42

## Discussion

In the context of gigantomastia, diverse surgical techniques have been employed by various authors, each presenting its unique considerations and outcomes. Some surgeons have shown a preference for the free nipple technique in gigantomastia cases, primarily aiming to address concerns related to NAC ischemia [17]. Others have chosen a combination of the free nipple technique with pedicle applications to enhance aesthetic results [7, 8, 16]. Furthermore, there has been a leaning toward pedicled techniques, incorporating various types of pedicles such as inferior, superomedial, and others [4, 9, 12–15]. Despite the array of techniques, concerns about NAC sensation, its aesthetic appearance, insufficient breast projection, potential NAC graft necrosis following the free nipple technique, and apprehensions regarding bottom-out deformity after the inferior pedicle technique have led many surgeons to increasingly favor superomedial or medial pedicles [3, 9, 11]. Hall-Findlay [3] succinctly summarized additional advantages of the medial pedicle, emphasizing preservation of the glandular structure, greater effectiveness in cases requiring more reduction in lateral fullness, and good preservation of NAC sensation.

The sensation and blood supply of the breast, along with the relationship of these anatomical aspects to different pedicle applications, were elucidated by Hall-Findlay and Shestak [5]. They emphasized that NAC sensation primarily originates from the anterolateral branch of the 4^th^ intercostal nerve, which sends superficial and deep branches into the subcutaneous tissue and over the pectoralis fascia, respectively. In most reduction mammoplasty cases, the superficial branch is severed, except for those in which a lateral pedicle is preferred. Conversely, the deep branch can be included within the superomedial or inferior pedicles if the integrity of the pectoralis fascia is preserved. Additionally, contributions from the branches of the 3rd and 5th intercostal nerves play a role in innervation of the NAC. The findings of Schreiber et al. are parallel to this anatomical information [2]. The authors evaluated Nahabedian’s reduction mammoplasty cases with different pedicles concerning postoperative NAC sensation. They categorized cases with excisions over 1000 g. as large-volume reduction mammaplasty and preferred the free nipple technique for excisions over 1500 g. The authors reported an average excision of 1.7 kg. For cases with the medial pedicled reduction technique, emphasizing the well-protected NAC sensation.

Hall-Findlay and Shestak [5] summarized breast vascularization in relation to breast pedicles as follows: the superficial branch of the lateral thoracic artery supplies the lateral pedicle; the branch of the internal mammary artery originating from the 2nd intercostal space supplies the superior, the 3rd branch supplies the medial, and the 4th branch supplies the inferior and central pedicles. The branch emerging from the 5th intercostal space and advancing to the breast fold contributes to the vascularization of the inferior pedicle. Veins other than the vena comitantes are superficial and generally drain in the superomedial direction. The authors stated that the vascular anatomy of the breast may vary among individuals and any necrosis of the NAC that may occur is more likely to be associated with a non-dominant blood supply rather than a mistake during surgery. They suggested that it is possible to thin the medial- and lateral-based pedicles because they are supplied by the superficial arterial system. However, they advocated the use of a full-thickness pedicle to protect the ducts and added that thinning of the upper part of the superomedial pedicle to facilitate its rotation is possible due to the superficial position of the second intercostal artery and veins. In light of this anatomical information, we marked the superficial veins in the preoperative drawings and tried to keep them within the superomedial pedicle, ensuring that the blood return from the pedicle was as robust as possible.

Details regarding flap preparation and inserting into position in breast reduction surgeries have been extensively discussed in numerous studies. Hall-Findlay’s endorsement of the medial pedicle technique underscored its ease of rotation to the desired position full thickness without folding [3]. However, in cases of gigantomastia with longer planned pedicles, maintaining a wide flap base for NAC vascular supply might necessitate a superomedial pedicle design, introducing concerns about bulkiness that could impact NAC vascularity after rotation. Landau et al. [6] argued that breast reduction with superomedial pedicle is safe for gigantomastia. Hinson et al. [9] shared their experiences with 31 gigantomastia cases with medial-superomedial (10%), medial (65%) and inferior (16%) pedicles. They preferred the inferior pedicle in cases with SN-N length over 40 cm and fold-nipple length less than 20 cm. The authors revealed that the flap technique, whether medial-superomedial, medial, or inferior, does not significantly impact complication rates. They noted that in cases where there are challenges with the insertion of the superomedial pedicle, excising the upper lateral segment of the flap (hence canceling the second intercostal artery) is a viable option in very large breasts. The authors did not encounter total NAC loss in any of their cases. Finger et al. [10] prepared the distal part of the superomedial flap with a thickness of 2 cm and increased the thickness of the flap toward the proximal part. The authors followed the method of keeping the base wider as the flap gets longer. Basaran et al. [8] were among those combining free nipple and pedicle techniques in gigantomastia cases to ensure NAC viability and provide sufficient breast volume. They also favored the superomedial pedicle for its easier rotation. To facilitate the insertion of the superomedial pedicle, the authors determined the Wise pattern’s vertical limb length as 6.5 and kept the pedicle length at 11 cm on average.

In designing the superomedial-based flaps, we adhered to the 1:3 ratio, maintaining a wide base and included the superficial venous system in the flap. We selectively thinned and elevated the distal part of the flap to aid rotation, considering the specific angle of divergence (80 degrees) and length of vertical limbs (6–8 cm) tailored to the pedicle length. We emphasize the importance of individualized flap design in gigantomastia cases, noting that as flap length increases, the rotation pivot point shifts distally, obviating the need for interventions like thinning the superolateral component of the flap base. Whether a thinner flap with a medial pedicle or a flap with a superomedial pedicle is safer requires further research, opening avenues for future exploration in this field.

We highlight the significance of anticipating and addressing circulatory issues in cases where a large pedicle design carrying the NAC is employed. Our observation of NAC ischemia in 3 out of 19 patients (15.79%), or 4 out of 38 breasts (10.53%), underscores the importance of both expecting circulatory problems and promptly detecting and effectively treating them. In instances of venous problems, we emphasize reducing flap pressure by alleviating edema, removing stitches, and ensuring improved oxygenation as crucial preventive measures.

We argue for the manageability of ischemia during surgery by converting the pedicled technique to the free nipple technique and addressing postoperative ischemia with an immediate and effective rescue protocol. This leads us to assert that considering the pedicled technique as the primary option in cases of advanced gigantomastia may not pose harm. However, we acknowledge the necessity of further research and comparative studies to solidify this perspective.

Comparing cases with and without NAC ischemia, the study found that factors such as age, BMI, smoking, weight, and SN-N distance did not significantly influence the results. However, we acknowledge the limitation of the small study population, emphasizing the rarity of large series in gigantomastia cases. Several strategies have been utilized to ensure proper vascularization of the NAC in cases of gigantomastia. The modifications to the Wise-pattern design may vary depending on the severity of gigantomastia or ptosis, aiming to support NAC circulation. One strategy involves extending the vertical arms of the Wise-pattern. Hinson et al. [9] determined that these lengths range from 10 to 11 cm. They also mentioned that these lengths may be further extended depending on the specific case. The authors also observed that patients who desire a very small breast size may choose the free nipple technique, while preserving more breast tissue through pedicled techniques can help maintain NAC vascularization, particularly for patients with a high BMI. Additionally, another precaution is to position the new NAC slightly below the mammary fold. In our cases, we implemented this approach, resulting in an average SN-N distance of 23 cm for our patients. Additionally, we noted a trend in the absence of problems in the last 13 cases with narrower Wise pattern angles and longer limbs, suggesting the need for a tailored Wise model for each patient. This underscores the importance of individualized approaches in gigantomastia surgeries.

Although we encountered NAC ischemia in the postoperative period in 4 breasts, we did not observe any seroma or serious wound healing problems. We think that we owe this situation to not using monopolar cautery during tissue excision and applying a drain for at least 1 week in each case. Although, it has been stated that the use of drains does not affect the complication rate in reduction mammaplasty cases [1, 9], we feel the use of drains for 1 week may be safer, especially in cases of gigantomastia. As a matter of fact, as we frequently observed in our gigantomastia cases that serous fluid drainage exceeds 30-40 cc per day for at least a week.

We acknowledge the potential benefits of HOT and VAT in managing NAC ischemia and fat necrosis. While the supportive effect of HOT on wound healing is widely accepted, its use in breast surgery in the literature is mostly related to the management of ischemic flaps after breast reconstructions; the application of this treatment in reduction mammaplasty and NAC-related complications appears to be even rarer [21–23]. Similarly, reports on the application of VAT, which is widely used in wound treatment, in NAC ischemia are relatively rare [20]. Although we express the opinion that more research is needed to determine the routine use of HOT and VAT in reduction mammaplasty, we think that these treatments can be added to the protocols to be used in NAC ischemia.

As previously mentioned, ischemia was diagnosed based on clinical observations during the early postoperative period in our cases. When it comes to the breast, the monitoring of postoperative flaps is primarily conducted in applications related to reconstruction [24, 25]. In an article regarding breast reconstruction using autologous tissue, Chang [24] stated that most researchers agree that clinical observation remains the gold standard for monitoring postoperative flaps. However, we believe that incorporating advanced diagnostic techniques, such as indocyanine green angiography, in the future could enable us to intervene earlier and more effectively in cases of ischemia [25, 26].

## Conclusion

In summary, our study encourages the consideration of the superomedial flap for gigantomastia treatment and highlights the importance of a comprehensive protocol, including measures to address postoperative complications. The potential roles of HOT and VAC in NAC-saving protocols are acknowledged, with the understanding that further research is necessary to solidify their routine use in reduction mammaplasty complications. The findings underscore the need for individualized approaches and continuous refinement of surgical techniques to enhance outcomes in gigantomastia cases.

1. The authors declare that they have no conflicts of interest to disclose.
2. This article does not contain any studies with human participants.
3. For this type of study, informed consent is not required.
